# Senescence: A DNA damage response and its role in aging and Neurodegenerative Diseases

**DOI:** 10.3389/fragi.2023.1292053

**Published:** 2024-03-21

**Authors:** Tejal Shreeya, Mohd Saifullah Ansari, Prabhat Kumar, Muskan Saifi, Ali A. Shati, Mohammad Y. Alfaifi, Serag Eldin I. Elbehairi

**Affiliations:** ^1^ Institute of Biophysics, Biological Research Center, Szeged, Hungary; ^2^ Doctoral School of Theoretical Medicine, University of Szeged, Szeged, Hungary; ^3^ Institute of Genetics, Biological Research Center, Szeged, Hungary; ^4^ Doctoral School of Biology, University of Szeged, Szeged, Hungary; ^5^ Institute of Physiology, Medical School, University of Pécs, Pécs, Hungary; ^6^ Centre for Neuroscience, University of Pécs, Pécs, Hungary; ^7^ S.S.V (PG) College, Hapur, Uttar Pradesh, India; ^8^ Biology Department, Faculty of Science, King Khalid University, Abha, Saudi Arabia

**Keywords:** DNA damage response, neurodegenerative diseases, neuroinflammation, neuronal death, SASP and senescence

## Abstract

Senescence is a complicated, multi-factorial, irreversible cell cycle halt that has a tumor-suppressing effect in addition to being a significant factor in aging and neurological diseases. Damaged DNA, neuroinflammation, oxidative stress and disrupted proteostasis are a few of the factors that cause senescence. Senescence is triggered by DNA damage which initiates DNA damage response. The DNA damage response, which includes the formation of DNA damage foci containing activated H2AX, which is a key factor in cellular senescence, is provoked by a double strand DNA break. Oxidative stress impairs cognition, inhibits neurogenesis, and has an accelerated aging effect. Senescent cells generate pro-inflammatory mediators known as senescence-associated secretory phenotype (SASP). These pro-inflammatory cytokines and chemokines have an impact on neuroinflammation, neuronal death, and cell proliferation. While it is tempting to think of neurodegenerative diseases as manifestations of accelerated aging and senescence, this review will present information on brain ageing and neurodegeneration as a result of senescence and DNA damage response.

## Introduction

Cellular senescence is a ubiquitous process and is a state of irreversible cell cycle arrest, induced by a variety of cellular stimuli such as DNA damage, telomere shortening/dysfunction, oncogenic activation and chromatin disruption. It is defined by cell cycle arrest in G1 or G2 phase and prevents the proliferation of damaged cell (Vicencio et al., 2008). Cellular senescence limits the replicative lifespan of cells and contributes to aging and age-related diseases. It is linked with both physiological and pathological conditions. The biological function of senescence is to eliminate undesirable cells, which is essentially comparable to apoptosis. It regulates embryonic development, tumor suppression and wound healing. Senescence is the main factor influencing aging. Senescent cell accumulation increases with aging due to it’s increased production and decreased clearance (Karin and Alon, 2021). Accumulation of senescent cells in the tissue, destroy the neighboring cells and leads to inflammation. They resist apoptosis and secrete persistent pro-inflammatory signals that are fatal to neighboring cells. Senescence acts as a protective barrier against tumor progression.

### Cause of senescence

DNA is under constant risk of damage by exogenous and endogenous damaging agents. The damage is triggered in response to various conditions such as DNA damage, oxidative stress, ionizing radiation, telomere shortening/damage, mitochondrial dysfunction, chromatin disruption, oncogene activation (Dodig et al., 2019). These triggers leads to telomeric and non-telomeric DNA damage which in turn activates a cellular defense mechanism known as the DNA damage response (DDR), where cell cycle progression is halted to allow repair mechanisms to rectify the errors (Jackson and Bartek, 2009a). Senescence is regulated by various essential molecules such as p53, p16^INK4a^ and Rb (Retinoblastoma). Altered mitochondrial function play an essential role in senescence. Reactive oxygen species (ROS) generated from mitochondria can affect cellular senescence by inducing persistent DNA damage response, thus stabilizing the senescence (Sharma et al., 2023). The principal cause of senescence is DNA damage which activates DNA damage response (DDR) and canonical p53-p21 pathway. Cell’s repair mechanisms when overwhelmed elicits senescence via p53 phosphorylation. The epigenetic alterations cause senescence via p16-Rb pathway. Senescent cells exhibit permanent cell cycle arrest regulated by p16^INK4A^ and p53-p21-Rb. Elevated expression of p53 upregulates the expression of p21, which arrests cell cycle. P16^INK4A^ arrests cell cycle by inhibiting CDK4 and CDK6, which inhibits cells entry to S-phase by the hyper-phosphorylation of Rb.

### Senescence associated secretory phenotype

Senescent cells secretes a complex pro-inflammatory mediators known as Senescence Associated Secretory Phenotype (SASP) which is a characteristic feature of senescent cells. SASP includes chemokines, cytokines and some growth factors. These inflammatory mediators cause inflammation and might also be crucial for the clearance of senescent cells by phagocytosis. SASP has paracrine and autocrine activities and it establishes an inflammatory microenvironment by modulating immune response, tissue remodeling, contributing to the surveillance and eventually leading to the elimination of senescent cells (Muñoz-Espín and Serrano, 2014; Gorgoulis et al., 2005). The pro-inflammatory mediators of SASP includes cytokines (e.g., IL-6, IL-8), chemokines (e.g., CXCL1, CXCL2), growth factors (e.g., TGF-β, VEGF), matrix metalloproteinases (e.g., MMP-3, MMP-9), and others (Coppé et al., 2008; Copp et al., 2010; Basisty et al., 2019). The JAK-STAT and NF-κB signaling pathways have emerged as a key regulators of cellular senescence and the SASP (Xu et al., 2015). The activation of this pathway amplifies the secretion of SASP factors, promoting inflammation and even tumorigenesis. Inhibition of this pathway could be used as a therapeutic target to combat the deleterious effects of senescence. DNA damage activates the NF-κB, which translocates to the nucleus and drives the expression of SASP factors (Chien et al., 2011). Persistent activation of NF-κB leads to the chronic secretion of pro-inflammatory factors that could be detrimental to the tissue milieu. A continuous feedback loop is established between NF-κB and the SASP, maintaining the state of senescence.

Senescence activates immune cells. SASP stimulates immune surveillance that result in the clearance of the senescent cells. SASP activation is regulated by NF-kB and CCAAT/enhancer binding protein B. SASP activation responds to hierarchical model where cytokines such as IL-1α and IL-1β signal via IL-1R, leading to a cascade of other cytokines and chemokines. The innate signaling pathway between TLR2 and A-SAAs initiates the SASP and shores up the cellular senescence. SASP components such as acute serum amyloids A1 and A2 (A-SAAs) are senescent associated DAMPs sensed by TLR2 after oncogenic induced senescence (OIS). TLR2 requires typical enhancers for their activation and is essential for both activation of p38MAPK and NF-κB during OIS. TLR2 signaling is crucial for SASP and cell cycle arrest and also plays a key role in the activation of p38MAPK and NF-kB signal transduction pathway (Hari et al., 2019).

The expression of TLR and release of DAMPs in many tumor cases suggests the existence of a crosstalk between them. TLR activation by DAMPs induces secretion of various cytokines within tumor environment. These cytokines modulate the interaction between tumor cells and immune cells thereby deciding the fate of the tumor. TLR-DAMP interaction has a dual role. On one hand DAMP-TLR activation, activates potent immunostimulants triggering anti-cancer response while on the other hand, it enhances immunosuppression and promotes angiogenesis and infiltration of suppressor cells which is a hallmark of cancer. The interaction of DAMPs with TLRs initiates an inflammatory cascade aiding tumor growth. As a result, control of cell cycle and contact inhibition is lost. Dying tumor cells release DAMPs which interact with TLRs expressed by the infiltrating inflammatory cells and tumor cells, regulating apoptosis of tumor cells thereby further release of DAMPs. Released DAMPs activate TLR signaling secreting more DAMPs and cytokines, thereby enhancing tumor cell death and leading to activation of pathways that result in tumor rejection and escape (Patidar et al., 2018). Activation of TLR2/6 induces senescence and leads to increased TLR2 and SASP expression (Mannarino et al., 2021).

SASP is associated with cellular senescence and it has both beneficial and detrimental effects. Though senescence and SASP has detrimental effect but their beneficial role cannot be neglected. Inducing senescence and SASP in cancer cells could be therapeutic strategy for the treatment of the disease. Upon senescence induction, cancer cells stop dividing and is recognized and eliminated by the immune system. SASP components such as growth factors promote tissue regeneration and repair injury or disease (Cuollo et al., 2020). Senolytics are drugs or compounds that target and eliminate senescent cells and clear harmful SASP while senomorphics abolishes the SASP phenotype without killing the senescent cells. Exploiting both of these could be a useful therapeutic strategy against cancer and age related neurodegenerative diseases (Chaib et al., 2022).

### Cellular modifications in senescent cells

It is fascinating to understand that cells have a specific mechanism that prevents the proliferation of damaged cell. Senescence being one of those fascinating mechanism. Senescent cells are stable, viable and metabolically active unlike the cells destined for apoptosis and autophagy. Cells undergoing senescence become larger and flatter due to the alterations in their cell membrane and cytoskeleton (MacIel-Barón et al., 2018) There is a disproportionate increase in the cytoplasm to nucleus ratio which may be a result of cytoskeletal rearrangements.

Senescence is associated with changes in cellular metabolism, proteostasis, elevated reactive oxygen species (ROS) production, mitochondrial dysfunction, lipofuscin accumulation, SASP production and upregulation of lysosomal enzyme- β-galactosidase thereby elevating the expression of senescence associated β-galactosidase (SA-βgal), which is a biomarker of senescence. Senescence also affects cellular metabolism and organelle function. Altered mitochondrial function elevates ROS generation in the senescent cell. Lipofuscin is the key feature of cellular senescence and is used for positive identification of senescent cells. Loss of nuclear Lamin B1 and HMGB1 is also detected in senescent cells. Besides all the cellular changes, there is an upregulation of cell survival pathways, including BCL-2. These phenotype are experienced by both mitotic and post-mitotic cells (Gorgoulis et al., 2019). The type of damage and the type of cell determine whether the cell enters senescence or apoptosis. Another feature of senescent cells is the accumulation of lipofuscin.

## Biomarkers of senescence

The major limitation in the field of senescence is the lack of single, universal, reliable biomarker to study senescence. The first and the most widely used biomarker is senescence associated β-galactosidase (SA-βgal) (Dimri et al., 1995). It is a colorimetric assay, detected by histochemical staining. This marker is absent in presenescent, quiescent and immortal cells (Evangelou et al., 2017).

P16 and p21 are cyclin dependent kinase inhibitor, governed by Rb and p53, often gets accumulated in senescent cells. They are used in the identification of senescent cells. Nuclear senescence-associated heterochromatin foci (SAHF) are also used to identify senescent cells, but they appear to be specific to the senescence program induced by activated oncogenes and DNA replication stressors (Di Micco et al., 2011). Phosphorylation of the core histone protein H2AX is the earliest response of the double stranded break in the DNA. This phosphorylation is mediated by ATM and ATR at the C-terminal of the histone protein at a conserved amino acid serine 139 (S139). The role of γH2AX is to recruit the associated protein needed for the DNA repair. Phosphorylated γH2AX is also used as a marker for DNA damage and senescence. Senescent markers and animal models have boosted our knowledge in understating senescence.

## DDR activation

Nuclear DNA is under constant threat of endogenous and exogenous agents. Rupture of DNA strands either single or double strand activate DNA damage response (DDR). DDR is a complex network of signaling pathways and molecular mechanisms, safeguards genomic integrity and orchestrates cellular responses to DNA lesions (Jackson and Bartek, 2009b). It is a crucial step in cellular senescence, triggered by DNA damage such as double-strand breaks (DSBs), telomere dysfunction, and oxidative damage. (17) When cells sustain DNA damage, DDR signaling pathways are engaged, triggering DNA repair mechanisms (d’Adda di Fagagna, 2008). The DDR pathway involves a cascade of processes such as DNA damage recognition, signal amplification, and activation of effector molecules, which in turn results in cell cycle arrest, DNA repair, or apoptosis (Zou and Elledge, 2003).

DDR activation initiation includes the recognition of DNA damage by sensor proteins, like the MRN complex (MRE11-RAD50-NBS1), PARP1 (poly (ADP-ribose) polymerase 1), and the Ku complex which detects DNA strand breaks (either single (SSBs) or double strand break (DSB)) and DNA crosslinks. Recognition of damaged DNA recruits sensor proteins and activates key transducers, including ATM (ataxia-telangiectasia mutated), ATR (ataxia-telangiectasia and Rad3-related), and DNA-PK (DNA-dependent protein kinase) (Zou and Elledge, 2003). These kinases phosphorylate downstream substrates, including histone H2AX (γH2AX), p53, and CHK1/CHK2, leading to the amplification and propagation of the DDR signal.

### p53-mediated DNA damage response and senescence

**FIGURE 1:**
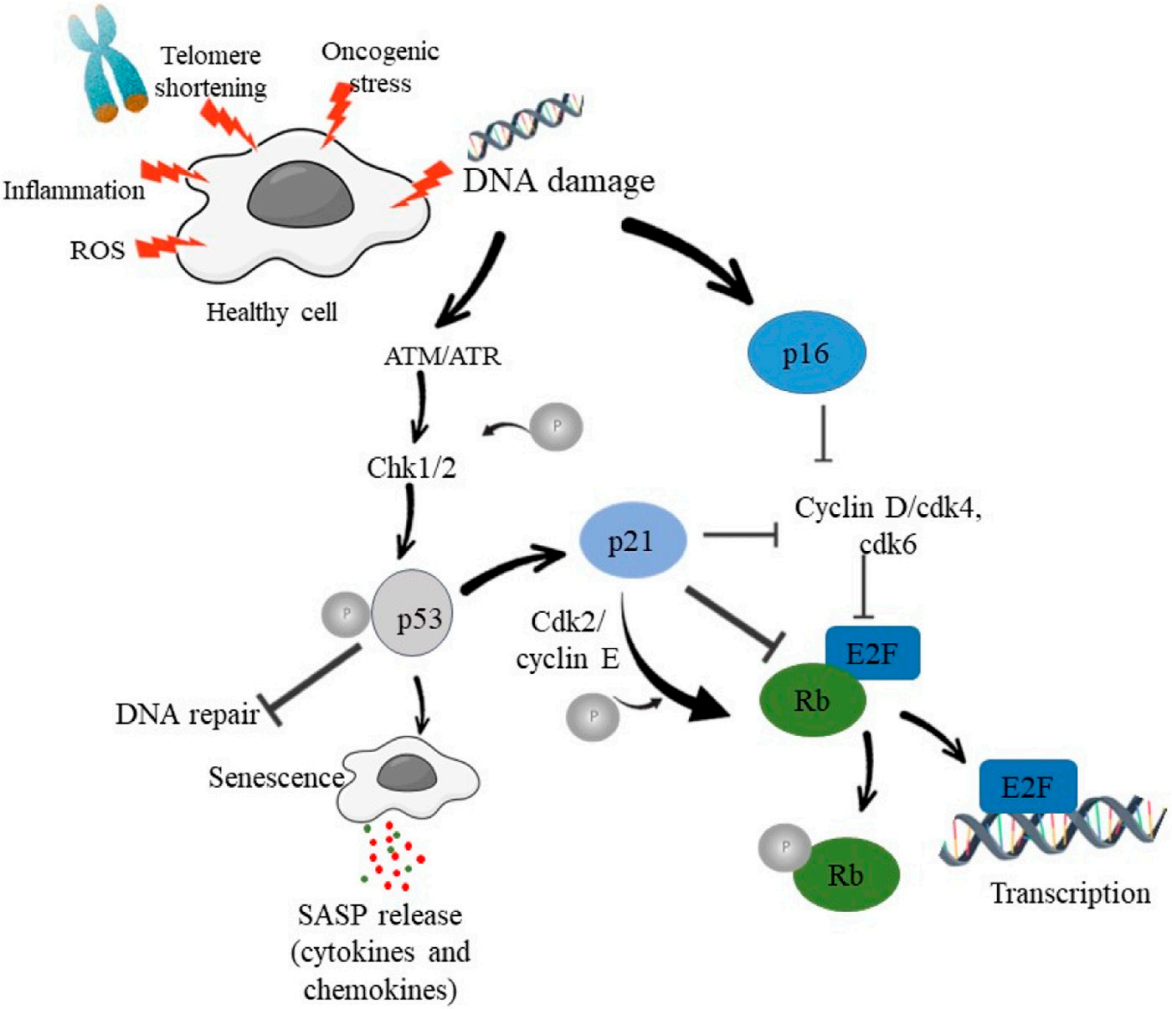
Schematic diagram showing DNA damage and activation of DDR in order to induce cell cycle arrest and senescence.

p53, a tumor suppressor protein is a key regulator of cellular responses to DNA damage. (16) p53 protein is known as “guardian of the genome”, regulating cellular responses to DNA damage, oncogenic stress, and other types of cellular stress (Lane, 1992). p53 plays a critical role in driving cellular senescence through the upregulation of specific target genes involved in cell cycle arrest and SASP (Gorgoulis et al., 2005) Activation of p53 leads to cell cycle arrest, DNA repair, apoptosis, and senescence, all aimed at preventing the propagation of damaged DNA and maintaining genomic stability (Vousden and Lane, 2007). Senescence acts as a protective mechanism by preventing the proliferation of damaged or potentially cancerous cells (Muñoz-Espín and Serrano, 2014). DDR activation stabilizes and activates tumor suppressor protein p53. Phosphorylation of p53 by ATM and CHK2 prevents its degradation, thereby enhancing its accumulation and its translocation to the nucleus. In the nucleus, p53 acts as transcription factor thereby regulating the expression of various genes involved in the process of cell cycle arrest, senescence and apoptosis (Velimezi et al., 2013). p53 activation triggers cell cycle arrest at the G1/S or G2/M checkpoints by upregulating the expression of p21 and other cyclin-dependent kinase inhibitors (CDKIs). The cell cycle arrest provides sufficient time for DNA repair and hence prevents damaged DNA from being passed on to the daughter cells. DDR activation may cause irreversible cell cycle halt, leading to cellular senescence, in circumstances of severe or persistent DNA damage (Tort et al., 2005). Figure 1 shows the schematic mechanism of p53 activation.

p53 acts as a transcription factor, post-translational modifications, such as phosphorylation, acetylation, ubiquitination, and methylation, modulate p53 stability and transcriptional activity (Jackson and Durocher, 2013). These modifications play a crucial role in fine-tuning p53-mediated DDR and senescence (Childs et al., 2015). p53 is regulated by regulators such as MDM2, ARF, and the E3 ligases which control p53 levels, stability, and nuclear localization, thereby modulating its transcriptional activity in response to DNA damage. Inactivation of p53 caused as a result of mutations, is a hallmark of various cancers. Loss of functional p53 compromises DDR and senescence, allowing cells with genomic instability to survive and proliferate thereby increasing the risk of tumor development (Vousden and Lane, 2007). p53-dependent senescence acts as a potent barrier to tumor formation, suppressing the growth of premalignant or cancerous cells. Restoration of p53 function or activation of senescence pathways holds therapeutic potential. Strategies aiming to reactivate p53 or induce senescence in cancer cells are being explored as potential therapeutic interventions. Combination therapies targeting DDR and senescence pathways show promising effect in enhancing the efficacy of conventional cancer treatments (Muñoz-fontela et al., 2016). Activated p53 induces cell cycle arrest allowing time for DNA repair before proceeding with cell division (Meek, 2015). This process prevents the propagation of damaged DNA to daughter cells (Vousden and Prives, 2009). p53 causes apoptosis in circumstances of severe DNA damage or irreversible genomic abnormalities, eradicating cells with weakened genetic material to stop the spread of mutations (Surova and Zhivotovsky, 2013).

### p16-mediated DNA damage response and senescence

DNA damage and the onset of cellular senescence are closely connected by p16 (Sherr and Mccormick, 2002). p16 provides a line of defense against the propagation of DNA mutations, which could otherwise lead to diseases such as cancer (Gil and Peters, 2006). p16 (p16^INK4A^) is a cyclin-dependent kinase inhibitor that regulates the cell cycle, an integral process of cell division and growth. It plays a pivotal role in the process of DDR, causes cell cycle arrest thereby promoting cellular senescence (Sharpless and Depinho, 1999; Sherr and Hughes, 2001). By blocking CDK4 and CDK6, p16^INK4A^ stops Rb from being phosphorylated. In its hypophosphorylated state, Rb binds to and inhibits E2F transcription factors, preventing the cell cycle’s passage from the G1 to the S phase. In order to promote persistent cell cycle arrest, the cell can induce senescence by raising the levels of p16^INK4A^. P16’s involvement in cell cycle regulation and senescence has far-reaching implications in human health. A dysfunctional p16 can lead to uncontrolled cell division and subsequent tumor development (Gil and Peters, 2006). Conversely, increased p16 expression has been linked with age-related pathologies, where senescent cells accumulate, leading to tissue dysfunction and aging (Campisi and d'Adda di Fagagna, 2007). For aging and age-related diseases, eliminating senescent cells or modulating their secretory phenotype (which is often pro-inflammatory and harmful to tissues) could be a promising avenue for research (Childs et al., 2014). A more in-depth understanding of its mechanisms and therapeutic potential will open doors for new interventions in combating various human diseases.

### Telomere shortening and DNA damage response in cellular senescence

Amongst all the cellular senescence inducers, telomere dysfunction is a well-established driver (Odds, 2012). Telomeres, a protective caps at the ends of chromosomes, are nucleoprotein structures composed of repetitive DNA sequences (TTAGGG in humans) and associated proteins, providing stability and protection for chromosome ends (Sullivan and Karlseder, 2010). Telomeres undergo continuous attrition due to “end replication problem” with each cell division (Shay and Wright, 2019). They lose their protective role when they become critically short, hence triggering a DNA damage signal. This trigger activates the DDR, leading to a permanent cell cycle arrest and the establishment of senescence (d’Adda di Fagagna, 2008). Telomere dysfunction can also arise independently of cell division and contribute to non-replicative senescence (Hewitt et al., 2012). Such insults lead to the exposure of telomeric DNA, inducing a persistent DDR and senescence-associated phenotype (Hewitt et al., 2012; Rodier and Campisi, 2011). Telomere dysfunction-induced senescence (TDIS) has been implicated in various age-related diseases, including cardiovascular disorders, neurodegenerative conditions, and cancer (Sullivan and Karlseder, 2010).

Telomeric dysfunction activates the DDR similarly as p53 and p16, through the activation of ATM (ataxia-telangiectasia mutated) and ATR (ataxia-telangiectasia and Rad3-related) kinases, which sense and signal telomere damage (Blackburn, 2001). These kinases phosphorylate downstream effectors, such as p53, CHK1, and CHK2, initiating cell cycle arrest, DNA repair, and senescence-associated secretory phenotype (SASP) activation. The JAK-STAT and NF-κB pathways are central to the regulation of cellular senescence and the associated inflammatory response. A better understanding of these mechanisms could open the way for therapeutic treatments to counteract the negative effects of cellular senescence in age-related illnesses and cancer.

### Role of LINE1 and KAT7 in cellular senescence

Senescence can be termed as a double-edged sword: as it acts as a defense mechanism against cancer, its accumulation is associated with aging and age-related diseases (Campisi, 2013). Emerging evidence suggests that Long Interspersed Element-1 (LINE-1) retrotransposons might play a pivotal role in the onset and progression of cellular senescence. Senescent cells have been shown to exhibit increased LINE-1 expression (De Cecco et al., 2019). Elevated LINE-1expression during senescence leads to the accumulation of LINE-1 RNAs and potential retrotransposition events. LINE-1 derepression in senescent cells initiates a type-I interferon (IFN-I) response, effectively acting as a cellular alarm to rising LINE-1 activity. This IFN-I response augments the senescence associated secretory phenotype (SASP), a significant source of chronic inflammation in aged tissues (Simon et al., 2019).

Several molecular players link LINE-1 activity with cellular senescence. A decline in Three Prime Repair Exonuclease 1 (TREX1), an exonuclease, during senescence leads to the accumulation of cytoplasmic LINE-1 cDNA (Tam et al., 2017). Reduced Retinoblastoma 1 (Rb1) expression in senescent cells can result in L1 derepression due to its role in binding and repressing LINE-1 elements (De Cecco et al., 2019). Elevated levels of FOXA1, a transcription factor in senescent cells, might enhance LINE-1 transcription. The interaction between LINE-1 activity and the inflammatory environment of aging, often termed “inflammaging”, has broad implications for understanding the biology of aging (Franceschi et al., 2018). The ties between LINE-1 derepression and increased inflammation could be a potential driver behind age-associated pathologies.

Given the link between LINE-1 activity and senescence, targeting LINE-1 with nucleoside reverse transcriptase inhibitors (NRTIs) might offer therapeutic benefits. In preclinical models, lamivudine (3 TC) has shown promise in dampening the inflammatory IFN-I response associated with L1 activity (De Cecco et al., 2019). In essence, LINE-1 elements, long dismissed as “junk DNA”, might hold significant clues to understanding the intricate dance of aging, inflammation, and cellular senescence. As we uncover more about LINE-1’s role in these processes, new avenues for therapeutic interventions in age-associated diseases might emerge.

Among the various molecular players implicated in the regulation of cellular senescence, KAT7 has emerged as a significant factor. KAT7, also known as HBO1 or MYST2, belongs to the MYST family of histone acetyl transferases (HATs). These enzymes play a crucial role in chromatin remodeling, gene transcription regulation, DNA replication, and DNA damage repair (Doyon and Côté, 2004). It is this ability of KAT7 to modulate chromatin structure and subsequently gene expression that ties it directly to the process of cellular senescence. One notable investigation found elevated levels of KAT7 in the liver tissues of older rats and humans. The study went on to demonstrate that silencing KAT7 in senescent cells could rejuvenate them, reversing the signs of aging (Wang W. et al., 2021). This revelation suggests that KAT7 is not just a marker of senescence but may also actively contribute to the onset and maintenance of the senescent state.

The molecular mechanism underlying KAT7’s role in senescence is believed to be associated with its histone acetylation function. KAT7, by influencing chromatin accessibility, can regulate the expression of genes that promote or inhibit cellular senescence. In particular, its role in the p16^INK4a^ and p21 pathways, has been highlighted (Narita et al., 2003). Elevated expression of KAT7 enhances the expression of these genes, leading to cell cycle arrest and entry into a senescent state. The discovery that KAT7 silencing can rejuvenate senescent cells hints at the possibility of harnessing this mechanism for anti-aging treatments. Moreover, since senescent cells have been implicated in various age-related pathologies, including neurodegenerative diseases, osteoarthritis, and cardiovascular diseases, targeting KAT7 could have broader medical implications (Baker et al., 2016).

### DDR and epigenetic changes in senescence

Cellular senescence is accompanied by alterations in DDR pathways and epigenetic modifications (Campisi and d'Adda di Fagagna, 2007; Hanahan and Weinberg, 2011). DDR not only influences the cell cycle arrest and DNA repair processes but also induces substantial epigenetic changes in senescent cells. DDR activation triggers alterations in DNA methylation patterns, histone modifications, and chromatin remodeling, resulting in the stable maintenance of the senescent state (Wang et al., 2009). One of the well-studied epigenetic modifications in senescence is DNA methylation, which involves the addition of a methyl group to cytosine residues predominantly at CpG dinucleotides. Global DNA hypomethylation and locus-specific DNA hypermethylation have been observed in senescent cells (Hernando-herraez et al., 2019). DNA hypomethylation leads to genomic instability and activation of transposable elements. On the other hand, DNA hypermethylation at specific gene promoters can result in transcriptional silencing of genes involved in cell cycle regulation, senescence, and tumor suppression (Liu et al., 2010). Senescence-associated alterations in histone modifications, such as methylation, acetylation and phosphorylation have also been reported (Feser and Tyler, 2011). These modifications can affect chromatin structure and gene accessibility.

The DDR signaling pathways directly influence epigenetic changes in senescence. DDR kinases such as ATM and ATR phosphorylate and activate histone methyltransferases and demethylases, leading to alterations in histone methylation patterns (Oh et al., 2014). DDR-induced activation of p53 can also impact DNA methylation patterns by regulating DNA methyltransferases and demethylases (Sharma et al., 2012) DNA methylation and histone modifications affects the expression and activity of DDR kinases, DNA repair genes, and cell cycle regulators, thereby influencing DDR efficacy and senescence-associated phenotype (Zhang et al., 2007; Zhang et al., 2005). The DDR, essential for maintaining genomic integrity, undergoes dysregulation during senescence, resulting in persistent DNA damage signals. Epigenetic changes, including DNA methylation, histone modifications, and non-coding RNAs, contribute to the stable maintenance of the senescent state. The interplay between DDR and epigenetic changes in senescence provides a fascinating area of research with potential therapeutic implications for aging and age-related diseases.

### DDR and senescence associated secretory phenotype (SASP): a complex interplay

SASP components recruit immune cells, facilitating the removal of the senescent cells, thus acting as a defense mechanism against potential tumors (Kang et al., 2011). They are also involved in tissue repair, potentially indicating a beneficial side to the SASP under certain contexts (Mosteiro et al., 2016). However, persistent secretion of SASP leads to chronic inflammation and tissue degradation, linking it to age-related diseases (Copp et al., 2010). There is an intricate interplay between DDR signaling, transcriptional factors, and epigenetic modifications (Table 1) and the activation and regulation of SASP. DDR activation, through the p38 mitogen-activated protein kinase (MAPK) pathway, drives the expression of SASP components. Additionally, transcription factors such as NF-κB, C/EBPβ, and STAT3 also plays crucial roles in the transcriptional upregulation of SASP genes (Freund et al., 2010). Emerging evidence suggests a positive feedback loop between DDR and SASP. DDR activation, driven by persistent DNA damage, triggers SASP secretion through the activation of SASP-inducing transcription factors. In turn, SASP components can further amplify DNA damage signaling by inducing DNA damage and senescence in neighboring cells, creating a senescence-associated pro-inflammatory microenvironment (Rodier and Campisi, 2011).

**TABLE 1:** Showing senescence phenotype and their cause of altercations.

Senescence phenotype	Cause of altercations
Loss of protein homeostasis. Nucleolar dysfunction. Accumulation of abnormal proteins (amyloid peptides and hyperphosphorylated tau)	Altered proteostasis
Cell cycle prolongation and re-entry	Cell cycle arrest
Aberrant phosphorylation of histones, changes in DNA methylation of AD critical genes. Mislocated chromatin organizing proteins and epigenetic regulators	Epigenetic modifications
Increased size, flat and irregular shape, changes in membrane composition	Morphological changes
Increased DNA damage and alterations in DDR.	Non-telomeric DNA damage and DNA repair mechanism
Increased ROS and altered mitochondrial structure and function that produces cellular changes associated with senescence	Oxidative stress and mitochondrial dysfunction
Microglia overactivation, enhanced release of pro-inflammatory cytokines and other SASP that aggravate amyloid and tau pathology	SASP
Telomeric DNA damage. Regarding telomere shortening controversial results	Telomeric DNA damage

## Senescence in Neurodegenerative Diseases

Neurodegenerative disease are characterized by chronic, progressive and pathological changes in the brain, such as neuronal death, abnormal aggregation of proteins and inflammation. Recent evidences suggest that the pathological changes in the neurodegenerative disease begins much ahead of the actual appearance of the symptoms. Prolonged exposure to stress, such as DNA damage may induce cellular senescence and contribute to the pathogenesis of the disease by altering the metabolism and affecting the gene expression. Recent studies have also suggested that there is an association between the length of telomeres and neurodegenerative disease. Longer telomere have proved to have a protective effect against dementia. Unfortunately, no association has been found in case of Parkinson’s disease.

### Alzheimer’s disease

Alzheimer’s disease (AD) is a chronic neurodegenerative condition that causes 60%–70% of dementia cases. Dementia, cognitive decline, amyloid plaques, neurofibrillary tangles (NFTs) of hyperphosphorylated tau proteins, and loss of neurons and synapses are the primary pathological hallmarks of AD. Nevertheless, there is substantial evidence that certain clinical events that occur years before the former are significant in forming amyloid plaques and NFTs. Processes such as oxidative stress, neuroinflammation are enhanced, and senescence brought on by DNA damage and disrupted proteostasis (Durst and Tropea, 2016; Wilcock, 2012). In recent years, research has explored the role of cellular senescence in the development and progression of Alzheimer’s disease, providing new avenues for potential therapeutic interventions. Senescent cells accumulate with age and are believed to contribute to age-related diseases, including Alzheimer’s disease. Alzheimer’s disease accumulates toxic protein aggregates in the brain, including amyloid-beta plaques and tau tangles. Recent studies have shown that cellular senescence plays a role in developing and accumulating these toxic protein aggregates. In 2018 Musi and their research team found that cellular senescence is associated with tau protein aggregation in the brain. The researchers combined genomic analysis with pharmacological interventions to induce senescence in neurons, which led to increased tau aggregation and neuronal dysfunction. Conversely, clearance of senescent cells reduced tau-dependent pathology. Cellular senescence is a critical factor in the development and progression of AD (Sipos et al., 2007). As evidenced by their increased SA-βgal expression, p53 expression, a mediator of cellular senescence, an increase in the release of SASP components, DNA damage, telomere attrition or damage, and senescence-like morphological changes, increased senescence is found in various cell types of AD brains, including astrocytes, microglia, and neurons (Caldeira et al., 2017). SA-βgal is normally present in low levels but its expression is elevated during senescence. Research has shown that SA-βgal expression is elevated in the brain regions affected by AD, specifically in the brains of AD patients (Copp et al., 2010). The tumor suppressor protein p53 is another crucial factor in senescence. p53 regulates cell growth and division in normal cells. It acts as a defense mechanism to prevent the proliferation of damaged cells. However, an overexpression of p53 is seen in senescent cells, leading to cell cycle arrest (Table 1) and the activation of pro-inflammatory pathways (Gorgoulis and Halazonetis, 2010).

Studies have suggested that the increased expression of SA-βgal and p53 in the brains of AD patients is linked to the accumulation of beta-amyloid plaques, a hallmark of the disease. Additionally, senescent cells with higher SA-βgal and p53 levels are more prevalent in plasma samples from AD patients and AD models of mice. These findings indicate that senescence plays a crucial role in the development of AD and that targeting senescent cells could be a possible therapeutic strategy for treating AD (Ihara et al., 2012). In addition, hyperphosphorylated tau protein, which is the hallmarks of AD, can cause glial cells to age. It has been hypothesized that cellular senescence plays a significant role in the etiopathology of AD as various early changes found in AD such as neuroinflammation, oxidative stress, DNA damage and changes in DNA repair, and altered proteostasis (Table 1) are linked to cellular senescence (Musi et al., 2018; Vermunt et al., 2019)According to research conducted *in vitro* on mouse neural stem cells, Aβ-42 oligomers cause senescent phenotype, which elevates the SA-βgal positive cells (He et al., 2013). These results are supported by several *in vivo* investigations using AD-induced animal models (Drummond and Wisniewski, 2017; Vitek et al., 2020; Tosca et al., 2023). SA-βgal decreased significantly in AD patient’s monocytes and lymphocytes compared to controls; this result was attributed to the upregulation of miR-128. The re-expression of multiple cell-cycle regulatory proteins in susceptible neurons lends weight to the hypothesis that abnormal cell cycle re-entry of the terminally differentiated post-mitotic neurons may play a significant role in the pathogenesis of AD (Tiribuzi et al., 2014; Vincent et al., 1996).

Specifically, the cyclin-dependent kinase inhibitor p21^CIP1^ is a critical player in disrupting the cell cycle in AD (Engidawork et al., 2001). The data still needs to be clarified, as some studies suggested elevated expression in the brains of AD patients compared to controls (Esteras et al., 2012; Yates et al., 2015) while other research found no appreciable difference. The findings from investigations on the peripheral blood lymphocytes and monocytes of AD patients, as well as from AD and tauopathy animal models, are equally intriguing. Neurons from AD patients and from animal models shows higher expression of p16^INK4a^. Increased p38MAPK activity has been observed in AD brains, lymphocytes, and in the cortex of AD mouse model (Esteras et al., 2012; Chang et al., 2000). Several crucial SASP components appear to be upregulated in AD, as p38MAPK is a significant regulator of SASP (Horstmann et al., 2010; Leake et al., 2000). Most notably, elevated levels of IL-6, IL-1, TGF, and TNF have been found in AD brain tissue, CSF and serum of AD patients. Also, elevated levels of the metalloproteinase MMP-1, MMP-3, and MMP-10 have been observed in AD patients (Horstmann et al., 2010; Sun et al., 2003; Wood et al., 1993). Due to abnormalities in methylation found in AD-affected brain regions and reports of aberrant DNA methylation patterns in multiple AD-associated genes, epigenetic changes appear to play a significant role in the etiology of the disease (Chung et al., 2019; Siddiqui et al., 2018). The hippocampus and lymphocytes of AD patients have also been found to have higher levels of phosphorylated histone γH2AX, indicating an active DNA damage response (Silva et al., 2014; Lord and Cruchaga, 2014). According to several lines of evidence, defects in autophagy and lysosomal dysfunction may have a role in the etiology and development of neurodegenerative illnesses, including AD (Qazi et al., 2018; Nixon, 2013).

Numerous studies have reported dysregulation in autophagic/lysosomal pathways in the development of AD, and the great majority of AD associated genes appear to be connected to these same pathways, which lends credence to this (Zare-shahabadi et al., 2015; Ihara et al., 2012) The interaction between autophagic/lysosomal failure and mitochondrial dysfunction, as well as their relationship to stress-induced premature senescence (SIPS) were the focus of recent studies (Tai et al., 2017; Yoon and Kim, 2016). All facets of mitochondrial function are compromised in AD, including aberrant dynamics and structure of the mitochondria and increased oxidative stress, which is already present in the very early stages of the disease and occurs before the major pathologic hallmarks, such as senile plaques and neurofibrillary tangles. As a result, mitochondria and lysosomes play a crucial role in the development of SIPS, albeit more investigation is required to determine how exactly they contribute to AD and senescence. In addition to neurons, all other cell types associated with AD disease have been shown to age. Astrocytes are essential contributors to the development and spread of the illness and, depending on various variables can have both advantageous and unfavorable effects (Gao et al., 2017; Chun and Lee, 2018).

Human astrocytes undergo senescence when exposed to Aβ oligomers, which also cause the synthesis of SASP constituents, including IL-6 and MMP-1, by activating the p38MAPK pathway (Erusalimsky, 2009). Additionally, astrocytes from AD hippocampus samples have been reported to contain elevated amounts of H2AX (Eitan et al., 2014). Even though the precise underlying mechanisms are still unknown, microglia have long been linked to the pathophysiology of AD (Conde and Streit, 2006; Hinterberger et al., 2017). Telomere shortening has been linked to replicative senescence in cultured microglial cells from AD patients (Flanary and Streit, 2004). Another cell component that plays a pivotal role in the complex mechanism underlying the senescence in AD is non-coding RNA. Non-coding RNAs play a crucial role in the complex processes of senescence in AD (Long et al., 2014). Although the precise cause of AD is not yet fully understood, there is growing evidence that non-coding RNAs, such as microRNAs (miRNAs) and long non-coding RNAs (lncRNAs), play a crucial role in regulating the mechanisms of neurodegeneration and aging in AD (Wang S. et al., 2021; Carrieri et al., 2012) miRNAs play a key role in regulating the expression of genes associated with synaptic function, neuroinflammation, and amyloid-beta processing. The dysregulation of specific miRNAs, including miR-132 and miR-146a, has been linked to the pathogenesis of AD (Hadar et al., 2018). It has been discovered that certain lncRNAs, such as BACE1-AS and BDNF-AS, play a significant role in the regulation of amyloid precursor protein (APP) metabolism and neuronal survival. Specifically, BACE1-AS serves as a miR-761 sponge, effectively inhibiting the degradation process facilitated by miR-761 and enhancing the expression of BACE1 in individuals affected by AD (Zeng et al., 2019). Another study conducted by Modarresiet al in 2011 and Zhang in 2018 which concludes that, the knockdown of BACE1-AS leads to a reduction in BACE1 and Aβ levels, resulting in the inhibition of tau protein phosphorylation in the hippocampus. This leads to enhanced memory and learning capabilities of SAMP8 mice (Lopez-Toledano et al., 2011; Zhang et al., 2018). These processes are of utmost importance in the context of AD (Carrieri et al., 2012). Neuroscientific research has shown that non-coding RNAs hold great potential as both therapeutic targets and diagnostic tools in the battle against Alzheimer’s disease. Unravelling the complex role of neurosciences in the process of senescence and neurodegeneration has the potential to reveal innovative approaches for early detection and intervention in this debilitating condition (García-Pérez et al., 2019; Pierouli et al., 2023).

Furthermore, dystrophic microglial cells that display morphological alterations suggestive of senescence have been linked to neuropathological characteristics of AD (Mosher and Wyss-Coray, 2014). *In vitro* aged rat microglia treated with Aβ oligomers develop a senescent phenotype, as seen by elevated levels of SA-βgal, IL-1β, TNF-α, and MMP-2. Neuroinflammation is a contributing factor to the acceleration of disease progression, resulting in cognitive impairment and damage to neurons. Transforming growth factor (TGF), conversely, initially has a neuroprotective function by facilitating cellular survival and tissue regeneration. However, when its control becomes disrupted, it can subsequently contribute to the development of fibrotic alterations. Tumor necrosis factor (TNF), a cytokine known for its pro-inflammatory properties, has been found to intensify neuroinflammation and play a role in the development of synaptic dysfunction Another study has revealed elevated levels of senescence-associated secretory phenotype proteins, including IL-6 and TGF-β in both cerebrospinal fluid (CSF) and plasma samples obtained from individuals diagnosed with Alzheimer’s disease (Caldeira et al., 2014; Si et al., 2021). Finally, it has been hypothesized that AD and telomere shortening are related (Caldeira et al., 2017; Caldeira et al., 2014). According to a sizable community-based longitudinal investigation, the telomere length of incident pure AD patients and cognitively healthy persons did not differ (Hinterberger et al., 2017).

### Parkinson’s disease

Parkinson’s disease (PD) is the most common movement disorder and the second most prevalent neurodegenerative disease after Alzheimer’s disease. It is a chronic neurodegenerative disease, characterized by the loss of dopaminergic neurons in the substantia nigra pars compacta of midbrain. It is also characterized by the aggregation of α-synuclein (α-syn) protein known as Lewy body formation. PD has a well-characterized motor symptoms (Si et al., 2021). The risk factor for neurodegenerative diseases is progressive age.

Senescence cells in CNS contribute to the neuropathology of various neurodegenerative diseases. Pre-symptomatic midbrain inflammation plays a crucial role in the pathology of PD. Cellular senescence triggers pro-inflammatory response- SASP, so senescence and SASP together are a strong contributing factor in the pathophysiology of PD. Despite decades of research, there is no cure for Parkinson’s disease, and the many subtleties of the pathology are still being worked out. The central nervous system plays an important role in neuroglia health and illness (Miller et al., 2022). Neurons, being the non-proliferative cells in the CNS, are also capable of undergoing cellular senescence. The dopaminergic (DA) neurons in PD has been noted to express various senescence markers (Sharma et al., 2023). Neuronal senescence has also been recognized to contribute to the “inflamm-aging” seen in PD.

In a recent study, it was found that α-syn aggregates triggers stress induced premature senescence in PD models. α-syn preformed fibrils (α-syn PFF) triggers cellular senescence in astrocytes and microglia and leads to their activation (Verma et al., 2021). Over activation of microglia has been detected in PD patients. Microglia, when activated produces inflammatory products which might contribute to the dopaminergic neuronal death in PD patients. Accumulation of α-syn leads to inflammatory activation of microglial cells. Interaction between α-syn and microglia suggest to play a role in the propagation of α-syn aggregation in PD. Senescence in endothelial cell (EC) is observed in aging and in diseased brain which leads to increased permeability of BBB and thereby leads to neurotoxicity in the brain. Senescent EC accumulation leads to increased SASP which further stimulate neuroinflammation. In PD, BBB becomes senescent causing impairment of BBB integrity (Russo and Riessland, 2022). Senescent astrocytes and SASP factors are elevated in Parkinsonian substantia nigra pars compacta. Elevated expression of p16^INK4a^, SASP factors are reported in AD patients. Reduced level of lamin B1 is an established senescent associated marker. Elevated presence of senescence in affected PD tissues.

It is still ambiguous how cellular senescence contributes to neurodegeneration in PD. SATB1, a Parkinson’s disease-related gene, guards against cellular senescence in dopaminergic neurons. In both humans and animals, SATB1 deficiency activates human stem cell-derived dopaminergic neurons. Dopamine neurons with SATB1 knockdown are required for cellular senescence. SATB1 influences the expression of the pro-senescence factor p21 in dopaminergic neurons (Riessland et al., 2019).

A common characteristic of many neurodegenerative disorders is an immune response. Parkinson’s patients have inflammation in the midbrain, however the underlying causes remain unknown. To disentangle the relationship between inflammation and senescent cells, it is critical to understand the potential origins of diverse midbrain cell types and how paracrine spreading of senescent cells amongst them may lead to observed local immune responses. The immune cell-mediated death of dopaminergic (DA) neurons in the midbrain of PD patients is the pro-inflammatory substance released by senescent cells (Russo and Riessland, 2022).

Elevated expression of p53 activity is observed in affected neurons of the PD patients as well as animal models. p53 activation, in response to various cellular stress, is associated with degeneration of dopaminergic neuron, which is accompanied by mitochondrial dysfunction, ROS production, and abnormal protein aggregation. The pathogenic p53 activates downstream, events to induce the degeneration of dopaminergic neurons. (103) Altered expression of p53 leads to neuronal death. Ho et al. (2019) hypothesize that the loss of dopaminergic neurons stimulated by the p53-p21 pathway via the G2019S LRRK2 mutation might be associated with cellular senescence, thereby promoting the accumulation of αSyn (Ho et al., 2019). In humans, the potential of both p16 and p21 expression as biomarkers of ageing and age-related diseases has previously been explored although not yet in the context of PD. Studies indicate that PD patients have lower mitochondrial DNA copies and longer telomeres. There is a correlation between the given medication and the telomeric length. Telomere length was shorter in patients with Parkinson’s disease, Parkinson’s disease dementia, and dementia with Lewy bodies compared to controls. There is also a correlation between the number of mitochondria in the blood and risk of PD (Asghar et al., 2022).

## Discussion

Understanding the intricate relationship between cellular senescence and DDR could open potential therapeutic avenues for age-related and neurodegenerative diseases. Targeting DDR components holds promise for modulating cellular senescence and regenerating aged tissues. Strategies aimed at depleting the detrimental effects of SASP could alleviate age-related inflammation and tissue dysfunction. Further investigations into the underlying mechanisms and therapeutic interventions targeting DDR and SASP may pave the way for novel strategies to promote healthy aging. Additionally, unraveling the complex crosstalk between DDR and epigenetic modifications may also provide insights into the fundamental mechanisms governing aging and senescence. DDR activation acts as a double-edged sword, promoting both beneficial effects by preventing the propagation of damaged DNA and detrimental effects through the establishment of senescence-associated phenotype. Further research is required to unravel the complex interplay between DDR and senescence and exploit this knowledge for therapeutic interventions in aging and age-related diseases. Despite major advances in the field of science, there are still significant gaps in understanding the cause of senescence, its relation with aging and its contribution to the neurodegenerative diseases.

## References

[B1] Asghar M. Odeh A. Fattahi A. J. Henriksson A. E. Miglar A. Khosousi S. (2022). Mitochondrial biogenesis, telomere length and cellular senescence in Parkinson’s disease and Lewy body dementia. Sci. Rep. 12 (1), 17578–17611. 10.1038/s41598-022-22400-z 36266468 PMC9584960

[B2] Baker D. J. Childs B. G. Durik M. Wijers M. E. Sieben C. J. Zhong J. (2016). Naturally occurring p16Ink4a -positive cells shorten healthy lifespan. Nature 530 (7589), 184–189. 10.1038/nature16932 26840489 PMC4845101

[B3] Basisty N. Kale A. Jeon O. Kuehnemann C. Payne T. Rao C. (2019). A proteomic atlas of senescence-associated secretomes for aging biomarker development. SSRN Electron. J., 1–26. 10.2139/ssrn.3380253 PMC6964821 31945054

[B4] Blackburn E. H. (2001). Switching and signaling at the telomere. Cell 106 (6), 661–673. 10.1016/S0092-8674(01)00492-5 11572773

[B5] Caldeira C. Cunha C. Vaz A. R. Falcão A. S. Barateiro A. Seixas E. (2017). Key aging-associated alterations in primary microglia response to beta-amyloid stimulation. Front. Aging Neurosci. 9 (August), 277–323. 10.3389/fnagi.2017.00277 28912710 PMC5583148

[B6] Caldeira C. Oliveira A. F. Cunha C. Vaz A. R. Falcão A. S. Fernandes A. (2014). Microglia change from a reactive to an age-like phenotype with the time in culture. Front. Cell. Neurosci. 8 (JUN), 152–216. 10.3389/fncel.2014.00152 24917789 PMC4040822

[B7] Campisi J. (2013). Aging, cellular senescence, and cancer. Annu. Rev. Physiol. 75, 685–705. 10.1146/annurev-physiol-030212-183653 23140366 PMC4166529

[B8] Campisi J. d'Adda di Fagagna F. (2007). Cellular senescence: when bad things happen to good cells. Nat. Rev. Mol. Cell Biol. 8, 729–740. 10.1038/nrm2233 17667954

[B9] Carrieri C. Cimatti L. Biagioli M. Beugnet A. Zucchelli S. Fedele S. (2012). Long non-coding antisense RNA controls Uchl1 translation through an embedded SINEB2 repeat. Nature 491 (7424), 454–457. 10.1038/nature11508 23064229

[B10] Chaib S. Tchkonia T. Kirkland J. L. (2022). Cellular senescence and senolytics: the path to the clinic. Nat. Med. 28 (8), 1556–1568. 10.1038/s41591-022-01923-y 35953721 PMC9599677

[B11] Chang B. D. Watanabe K. Broude E. V. Fang J. Poole J. C. Kalinichenko T. V. (2000). Effects of p21Waf1/Cip1/Sdi1 on cellular gene expression: implications for carcinogenesis, senescence, and age-related diseases. Proc. Natl. Acad. Sci. U. S. A. 97 (8), 4291–4296. 10.1073/pnas.97.8.4291 10760295 PMC18232

[B12] Chien Y. Scuoppo C. Wang X. Fang X. Balgley B. Bolden J. E. (2011). Control of the senescence-associated secretory phenotype by NF- k B promotes senescence and enhances chemosensitivity. Genes Dev. 25, 2125–2136. 10.1101/gad.17276711.and 21979375 PMC3205583

[B13] Childs B. G. Baker D. J. Kirkland J. L. Campisi J. Van Deursen J. M. (2014). Senescence and apoptosis: dueling or complementary cell fates. EMBO Rep. 15 (11), 1139–1153. 10.15252/embr.201439245 25312810 PMC4253488

[B14] Childs B. G. Durik M. Baker D. J. Van Deursen J. M. (2015). Cellular senescence in aging and age-related disease: from mechanisms to therapy. Nat. Med. 21 (12), 1424–1435. 10.1038/nm.4000 26646499 PMC4748967

[B15] Chun H. Lee C. J. (2018). Reactive astrocytes in Alzheimer’s disease: a double-edged sword. Neurosci. Res. 126, 44–52. 10.1016/j.neures.2017.11.012 29225140

[B16] Chung K. M. Hernández N. Sproul A. A. Yu W. H. (2019). Alzheimer’s disease and the autophagic-lysosomal system. Neurosci. Lett. 697, 49–58. 10.1016/j.neulet.2018.05.017 29758300

[B17] Conde J. R. Streit W. J. (2006). Microglia in the aging brain. J. Neuropathol. Exp. Neurol. 65 (3), 199–203. 10.1097/01.jnen.0000202887.22082.63 16651881

[B18] Copp J. Desprez P. Krtolica A. Campisi J., (2010). The senescence-associated secretory phenotype: the dark side of tumor suppression. Annu. Rev. Pathol. 5, 99–118. 10.1146/annurev-pathol-121808-102144 20078217 PMC4166495

[B19] Coppé J. P. Patil C. K. Rodier F. Sun Y. Muñoz D. P. Goldstein J. (2008). Senescence-associated secretory phenotypes reveal cell-nonautonomous functions of oncogenic RAS and the p53 tumor suppressor. PLoS Biol. 6 (12), 2853–2868. 10.1371/journal.pbio.0060301 19053174 PMC2592359

[B20] Cuollo L. Antonangeli F. Santoni A. Soriani A. (2020). The senescence-associated secretory phenotype (Sasp) in the challenging future of cancer therapy and age-related diseases. Biol. (Basel) 9 (12), 485–516. 10.3390/biology9120485 PMC7767554 33371508

[B21] d’Adda di Fagagna F. (2008). Living on a break: cellular senescence as a DNA-damage response. Nat. Rev. cancer cancer 8, 512–522. 10.1038/nrc2440 18574463

[B22] De Cecco M. Ito T. Petrashen A. P. Elias A. E. Skvir N. J. Criscione S. W. (2019). L1 drives IFN in senescent cells and promotes age-associated inflammation. Nature 566 (7742), 73–78. 10.1038/s41586-018-0784-9 30728521 PMC6519963

[B23] Di Micco R. Sulli G. Dobreva M. Liontos M. Botrugno O. A. Gargiulo G. (2011). Interplay between oncogene-induced DNA damage response and heterochromatin in senescence and cancer. Nat. Cell Biol. 13 (3), 292–302. 10.1038/ncb2170 21336312 PMC3918344

[B24] Dimri G. P. Lee X. Basile G. Acosta M. Scott G. Roskelley C. (1995). A biomarker that identifies senescent human cells in culture and in aging skin *in vivo* . Proc. Natl. Acad. Sci. U. S. A. 92 (20), 9363–9367. 10.1073/pnas.92.20.9363 7568133 PMC40985

[B25] Dodig S. Čepelak I. Pavić I. (2019). Hallmarks of senescence and aging. Biochem. Medica 29 (3), 030501–030515. 10.11613/BM.2019.030501 PMC6610675 31379458

[B26] Doyon Y. Côté J. (2004). The highly conserved and multifunctional NuA4 HAT complex. Curr. Opin. Genet. Dev. 14 (2), 147–154. 10.1016/j.gde.2004.02.009 15196461

[B27] Drummond E. Wisniewski T. (2017). Alzheimer’s disease: experimental models and reality. Acta Neuropathol. 133 (2), 155–175. 10.1007/s00401-016-1662-x 28025715 PMC5253109

[B28] Durst F. Tropea C. (2016). The amyloid hypothesis of Alzheimer’s disease at 25 years. EMBO Mol. Med. 8 (6), 595–608. 10.15252/emmm.201606210 27025652 PMC4888851

[B29] Eitan E. Hutchison E. R. Mattson M. P. (2014). Telomere shortening in neurological disorders: an abundance of unanswered questions. Trends Neurosci. 37 (5), 256–263. 10.1016/j.tins.2014.02.010 24698125 PMC4008659

[B30] Engidawork E. Gulesserian T. Seidl R. Cairns N. Lubec G. (2001). Expression of apoptosis related proteins in brains of patients with Alzheimer’s disease. Neurosci. Lett. 303 (2), 79–82. 10.1016/S0304-3940(01)01618-4 11311497

[B31] Erusalimsky J. D. (2009). Vascular endothelial senescence: from mechanisms to pathophysiology. J. Appl. Physiol. 106 (1), 326–332. 10.1152/japplphysiol.91353.2008 19036896 PMC2636933

[B32] Esteras N. Bartolomé F. Alquézar C. Antequera D. Muñoz Ú. Carro E. (2012). Altered cell cycle-related gene expression in brain and lymphocytes from a transgenic mouse model of Alzheimer’s disease [amyloid precursor protein/presenilin 1 (PS1)]. Eur. J. Neurosci. 36 (5), 2609–2618. 10.1111/j.1460-9568.2012.08178.x 22702220

[B33] Evangelou K. Lougiakis N. Rizou S. V. Kotsinas A. Kletsas D. Muñoz-Espín D. (2017). Robust, universal biomarker assay to detect senescent cells in biological specimens. Aging Cell 16 (1), 192–197. 10.1111/acel.12545 28165661 PMC5242262

[B34] Feser J. Tyler J. (2011). Chromatin structure as a mediator of aging. FEBS Lett. 585 (13), 2041–2048. 10.1016/j.febslet.2010.11.016 21081125 PMC3988783

[B35] Flanary B. E. Streit W. J. (2004). Progressive telomere shortening occurs in cultured rat microglia, but not astrocytes. Glia 45 (1), 75–88. 10.1002/glia.10301 14648548

[B36] Franceschi C. Garagnani P. Parini P. Giuliani C. Santoro A. (2018). Inflammaging: a new immune–metabolic viewpoint for age-related diseases. Nat. Rev. Endocrinol. 14 (10), 576–590. 10.1038/s41574-018-0059-4 30046148

[B37] Freund A. V Orjalo A. Desprez P. Campisi J. (2010). Inflammatory networks during cellular senescence: causes and consequences. Trends Mol. Med. 16 (5), 238–246. 10.1016/j.molmed.2010.03.003 20444648 PMC2879478

[B38] Gao J. Wang L. Liu J. Xie F. Su B. Wang X. (2017). Abnormalities of mitochondrial dynamics in neurodegenerative diseases. Antioxidants 6, 25–32. 10.3390/antiox6020025 28379197 PMC5488005

[B39] García-Pérez I. Fang Y. Guo Y. Hong W. Tu J. Wei W. (2019). The emerging role of long non-coding RNAs in tumor-associated macrophages. J. Cancer 10 (26), 6738–6746. 10.7150/jca.35770 31777603 PMC6856883

[B40] Gil J. Peters G. (2006). Regulation of the INK4b – ARF – INK4a tumour suppressor locus: all for one or one for all. Nat. Rev. Mol. Cell Biol. 7 (September), 667–677. 10.1038/nrm1987 16921403

[B41] Gorgoulis V. Adams P. D. Alimonti A. Bennett D. C. Bischof O. Bishop C. (2019). Perspective cellular senescence: defining a path forward. Cell 179, 813–827. 10.1016/j.cell.2019.10.005 31675495

[B42] Gorgoulis V. G. Halazonetis T. D. (2010). Oncogene-induced senescence: the bright and dark side of the response. Curr. Opin. Cell Biol. 22 (6), 816–827. 10.1016/j.ceb.2010.07.013 20807678

[B43] Gorgoulis V. G. Pratsinis H. Zacharatos P. Demoliou C. Sigala F. Asimacopoulos P. J. (2005). p53-Dependent ICAM-1 overexpression in senescent human cells identified in atherosclerotic lesions. Lab. Invest 502–511. 10.1038/labinvest.3700241 15711569

[B44] Hadar A. Milanesi E. Walczak M. Puzianowska-Kuźnicka M. Kuźnicki J. Squassina A. (2018). SIRT1, miR-132 and miR-212 link human longevity to Alzheimer’s Disease. Sci. Rep. 8 (1), 8465–8510. 10.1038/s41598-018-26547-6 29855513 PMC5981646

[B45] Hanahan D. Weinberg R. A. (2011). Hallmarks of cancer: the next generation. Cell 144 (5), 646–674. 10.1016/j.cell.2011.02.013 21376230

[B46] Hari P. Millar F. R. Tarrats N. Birch J. Quintanilla A. Rink C. J. (2019). The innate immune sensor Toll-like receptor 2 controls the senescence-associated secretory phenotype. Sci. Adv. 5 (6), eaaw0254–15. 10.1126/sciadv.aaw0254 31183403 PMC6551188

[B47] He N. Jin W. L. Lok K. H. Wang Y. Yin M. Wang Z. J. (2013). Amyloid-β(1-42) oligomer accelerates senescence in adult hippocampal neural stem/progenitor cells via formylpeptide receptor 2. Cell Death Dis. 4 (11), 9244–e1010. 10.1038/cddis.2013.437 PMC3847315 24263098

[B48] Hernando-herraez I. Evano B. Stubbs T. Commere P. H. Jan Bonder M. Clark S. (2019). Ageing affects DNA methylation drift and transcriptional cell-to-cell variability in mouse muscle stem cells. Nat. Commun. 10 (–11), 4361. 10.1038/s41467-019-12293-4 31554804 PMC6761124

[B49] Hewitt G. Jurk D. Marques F. D. Correia-Melo C. Hardy T. Gackowska A. (2012). Telomeres are favoured targets of a persistent DNA damage response in ageing and stress-induced senescence. Nat. Commun. 3, 708. 10.1038/ncomms1708 22426229 PMC3292717

[B50] Hinterberger M. Fischer P. Huber K. Krugluger W. Zehetmayer S. (2017). Leukocyte telomere length is linked to vascular risk factors not to Alzheimer’s disease in the VITA study. J. Neural Transm. 124 (7), 809–819. 10.1007/s00702-017-1721-z 28393276

[B51] Ho D. H. Seol W. Son I. (2019). Upregulation of the p53-p21 pathway by G2019S LRRK2 contributes to the cellular senescence and accumulation of α-synuclein. Cell Cycle 18 (4), 467–475. 10.1080/15384101.2019.1577666 30712480 PMC6422450

[B52] Horstmann S. Budig L. Gardner H. Koziol J. Deuschle M. Schilling C. (2010). Matrix metalloproteinases in peripheral blood and cerebrospinal fluid in patients with Alzheimer’s disease. Int. Psychogeriatrics 22 (6), 966–972. 10.1017/S1041610210000827 20561382

[B53] Ihara Y. Morishima-Kawashima M. Nixon R. (2012). The ubiquitin-proteasome system and the autophagic-lysosomal system in Alzheimer disease. Cold Spring Harb. Perspect. Med. 2, a006361. 10.1101/cshperspect.a006361 22908190 PMC3405832

[B54] Jackson S. P. Bartek J. (2009a). The DNA-damage response in human biology and disease. Nature 461 (7267), 1071–1078. 10.1038/nature08467 19847258 PMC2906700

[B55] Jackson S. P. Bartek J. (2009b). The DNA-damage response in human biology and disease. Biol. Dis. 461 (October), 1071–1078. 10.1038/nature08467 PMC2906700 19847258

[B56] Jackson S. P. Durocher D. (2013). Regulation of DNA damage responses by ubiquitin and SUMO. Mol. Cell 49 (5), 795–807. 10.1016/j.molcel.2013.01.017 23416108

[B57] Kang T. Yevsa T. Woller N. Hoenicke L. Wuestefeld T. Dauch D. (2011). Senescence surveillance of pre-malignant hepatocytes limits liver cancer development. Nature 479 (7374), 547–551. 10.1038/nature10599 22080947

[B58] Karin O. Alon U. (2021). Senescent cell accumulation mechanisms inferred from parabiosis. GeroScience 43 (1), 329–341. 10.1007/s11357-020-00286-x 33236264 PMC8050176

[B59] Lane D. P. (1992). P53, Guardian of the genome. Nature 358, 15–16. 10.1038/358015a0 1614522

[B60] Leake A. Morris C. M. Whateley J. (2000). Brain matrix metalloproteinase 1 levels are elevated in Alzheimer’s disease. Neurosci. Lett. 291 (3), 201–203. 10.1016/S0304-3940(00)01418-X 10984641

[B61] Liu L. Luo G. Z. Yang W. Zhao X. Zheng Q. Lv Z. (2010). Activation of the imprinted dlk1-dio3 region correlates with pluripotency levels of mouse stem cells * □,” J. Biol. Chem. 285, 25, 19483–19490. 10.1074/jbc.M110.131995 20382743 PMC2885227

[B62] Long J. M. Ray B. Lahiri D. K. (2014). MicroRNA-339-5p down-regulates protein expression of β-site amyloid precursor protein-cleaving enzyme 1 (BACE1) in human primary brain cultures and is reduced in brain tissue specimens of Alzheimer disease subjects. J. Biol. Chem. 289 (8), 5184–5198. 10.1074/jbc.M113.518241 24352696 PMC3931075

[B63] Lopez-Toledano M. A. Modarresi F. Faghihi M. A. Patel N. S. Sahagan B. G. Wahlestedt C. (2011). Knockdown of BACE1-AS nonprotein-coding transcript modulates beta-amyloid-related hippocampal neurogenesis. Int. J. Alzheimers. Dis. 2011, 929042. 10.4061/2011/929042 21785702 PMC3139208

[B64] Lord J. Cruchaga C. (2014). The epigenetic landscape of Alzheimer’s disease. Nat. Neurosci. 17 (9), 1138–1140. 10.1038/nn.3792 25157507 PMC5472058

[B65] MacIel-Barón L. Á. Moreno-Blas D. Morales-Rosales S. L. González-Puertos V. Y. López-Díazguerrero N. E. Torres C. (2018). Cellular senescence, neurological function, and redox state. Antioxidants Redox Signal 28 (18), 1704–1723. 10.1089/ars.2017.7112 28467755

[B66] Mannarino M. Cherif H. Li L. Sheng K. Rabau O. Jarzem P. (2021). Toll-like receptor 2 induced senescence in intervertebral disc cells of patients with back pain can be attenuated by o-vanillin. Arthritis Res. Ther. 23 (1), 117–214. 10.1186/s13075-021-02504-z 33863359 PMC8051055

[B67] Meek D. W. (2015). Regulation of the p53 response and its relationship to cancer. Biochem. J. 469, 325–346. 10.1042/BJ20150517 26205489

[B68] Miller S. J. Campbell C. E. Jimenez-Corea H. A. Wu G. H. Logan R. (2022). Neuroglial senescence, α-synucleinopathy, and the therapeutic potential of senolytics in Parkinson’s disease. Front. Neurosci. 16 (April), 824191–824220. 10.3389/fnins.2022.824191 35516803 PMC9063319

[B69] Mosher K. I. Wyss-Coray T. (2014). Microglial dysfunction in brain aging and Alzheimer’s disease. Biochem. Pharmacol. 88 (4), 594–604. 10.1016/j.bcp.2014.01.008 24445162 PMC3972294

[B70] Mosteiro L. Pantoja C. Alcazar N. Marión R. M. Chondronasiou D. Rovira M. (2016). Tissue damage and senescence provide critical signals for cellular reprogramming *in vivo* . Science 354, aaf4445-6315. 10.1126/science.aaf4445 27884981

[B71] Muñoz-Espín D. Serrano M. (2014). Cellular senescence: from physiology to pathology. Nat. Rev. Mol. Cell Biol. 15 (7), 482–496. 10.1038/nrm3823 24954210

[B72] Muñoz-fontela C. Mandinova A. Aaronson S. A. Lee S. W. (2016). Emerging roles of p53 and other tumour-suppressor genes in immune regulation. Nat. Publ. Gr. 16, 741–750. 10.1038/nri.2016.99 PMC5325695 27667712

[B73] Musi N. Valentine J. M. Sickora K. R. Baeuerle E. Thompson C. S. Shen Q. (2018). Tau protein aggregation is associated with cellular senescence in the brain. Aging Cell 17 (6), e12840. 10.1111/acel.12840 30126037 PMC6260915

[B74] Narita M. Nũnez S. Heard E. Narita M. Lin A. W. Hearn S. A. (2003). Rb-mediated heterochromatin formation and silencing of E2F target genes during cellular senescence. Cell 113 (6), 703–716. 10.1016/S0092-8674(03)00401-X 12809602

[B75] Nixon R. A. (2013). The role of autophagy in neurodegenerative disease. Nat. Med. 19 (8), 983–997. 10.1038/nm.3232 23921753

[B76] Odds S. (2012). Too toxic to ignore.

[B77] Oh J. Lee Y. D. Wagers A. J. (2014). Stem cell aging: mechanisms, regulators and therapeutic opportunities. Nat. Publ. Gr. 20, 870–880. 10.1038/nm.3651 PMC4160113 25100532

[B78] Patidar A. Selvaraj S. Sarode A. Chauhan P. Chattopadhyay D. Saha B. (2018). DAMP-TLR-cytokine axis dictates the fate of tumor. Cytokine, 104. 114–123. 10.1016/j.cyto.2017.10.004 29032985

[B79] Pierouli K. Papakonstantinou E. Papageorgiou L. Diakou I. Mitsis T. Dragoumani K. (2023). Role of non-coding RNAs as biomarkers and the application of omics technologies in Alzheimer’s disease (Review). Int. J. Mol. Med. 51 (1), 5–11. 10.3892/ijmm.2022.5208 36453246 PMC9747195

[B80] Qazi T. J. Quan Z. Mir A. Qing H. (2018). Epigenetics in alzheimer’s disease: perspective of DNA methylation. Mol. Neurobiol. 55 (2), 1026–1044. 10.1007/s12035-016-0357-6 28092081

[B81] Riessland M. Kolisnyk B. Kim T. W. Cheng J. Ni J. Pearson J. A. (2019). Loss of SATB1 induces p21-dependent cellular senescence in post-mitotic dopaminergic neurons. Cell Stem Cell 25 (4), 514–530. 10.1016/j.stem.2019.08.013 31543366 PMC7493192

[B82] Rodier F. Campisi J. (2011). Four faces of cellular senescence. Four faces Cell. senescence 192 (4), 547–556. 10.1083/jcb.201009094 PMC3044123 21321098

[B83] Russo T. Riessland M. (2022). Age-related midbrain inflammation and senescence in Parkinson’s disease,” Front. Aging Neurosci. 14, 917797–7. 10.3389/fnagi.2022.917797 35721008 PMC9204626

[B84] Sharma A. Singh K. Almasan A. (2012), Chapter 40 histone H2AX phosphorylation: a marker for DNA damage. Methods Mol. Biol. 920, 613–626. 10.1007/978-1-61779-998-3_40 22941631

[B85] Sharma K. Sarkar J. Trisal A. Ghosh R. Dixit A. Singh A. K. (2023). Targeting mitochondrial dysfunction to salvage cellular senescence for managing neurodegeneration. Adv. Protein Chem. Struct. Biol. 136, 309–337. 10.1016/bs.apcsb.2023.02.016 37437982

[B86] Sharpless N. E. Depinho R. A. (1999). The INK4A/ARF locus and its two gene products. Curr. Opin. Genet. Dev. 4, 22–30. 10.1016/s0959-437x(99)80004-5 10072356

[B87] Shay J. W. Wright W. E. (2019). Telomeres and telomerase: three decades of progress. Nat. Rev. Genet. 20 (May), 299–309. 10.1038/s41576-019-0099-1 30760854

[B88] Sherr C. J. Hughes H. (2001). The ink4a/arf network in tumour suppression. Nat. Rev. Mol. Cell Biol. 2, 1–7. 10.1038/35096061 11584300

[B89] Sherr C. J. Mccormick F. (2002). The RB and p53 pathways in cancer. Cancer Cell 2, 103–112. 10.1016/s1535-6108(02)00102-2 12204530

[B90] Si Z. Sun L. Wang X. (2021). Evidence and perspectives of cell senescence in neurodegenerative diseases. Biomed. Pharmacother. 137, 111327. 10.1016/j.biopha.2021.111327 33545662

[B91] Siddiqui M. S. Francois M. Hecker J. Faunt J. Fenech M. F. Leifert W. R., “γH2AX is increased in peripheral blood lymphocytes of Alzheimer’s disease patients in the South Australian Neurodegeneration, Nutrition and DNA Damage (SAND) study of aging,” Mutat. Res. - Genet. Toxicol. Environ. Mutagen., vol. 829-830, 6–18, no. pp, 2018. 10.1016/j.mrgentox.2018.03.001 29704994

[B92] Silva A. R. T. Santos A. C. F. Farfel J. M. Grinberg L. T. Ferretti R. E. L. Campos A. H. J. F. M. (2014). Repair of oxidative DNA damage, cell-cycle regulation and neuronal death may influence the clinical manifestation of Alzheimer’s disease. PLoS One 9 (6), e99897. 10.1371/journal.pone.0099897 24936870 PMC4061071

[B93] Simon M. Van Meter M. Ablaeva J. Ke Z. Gonzalez R. S. Taguchi T. (2019). LINE1 derepression in aged wild type and SIRT6 deficient mice drives inflammation. Cell Metab. 29 (4), 871–885. 10.1016/j.cmet.2019.02.014 30853213 PMC6449196

[B94] Sipos E. Kurunczi A. Kasza A. Horváth J. Felszeghy K. Laroche S. (2007). Beta-amyloid pathology in the entorhinal cortex of rats induces memory deficits: implications for Alzheimer's disease. Neuroscience 147 (1), 28–36. 10.1016/j.neuroscience.2007.04.011 17499931

[B95] Sullivan R. J. O. Karlseder J. (2010). Telomeres: protecting chromosomes against genome instability. Nat. Rev. Mol. Cell Biol. 11, 171–181. 10.1038/nrm2848 20125188 PMC2842081

[B96] Sun A. Liu M. Nguyen X. V. Bing G. (2003). p38 MAP kinase is activated at early stages in Alzheimer’s disease brain. Exp. Neurol. 183 (2), 394–405. 10.1016/S0014-4886(03)00180-8 14552880

[B97] Surova O. Zhivotovsky B. (2013). Various modes of cell death induced by DNA damage. Oncogene 32, 3789–3797. 10.1038/onc.2012.556 23208502

[B98] Tai H. Wang Z. Gong H. Han X. Zhou J. Wang X. (2017). Autophagy impairment with lysosomal and mitochondrial dysfunction is an important characteristic of oxidative stress-induced senescence. Autophagy 13 (1), 99–113. 10.1080/15548627.2016.1247143 27791464 PMC5240829

[B99] Tiribuzi R. Crispoltoni L. Porcellati S. Di Lullo M. Florenzano F. Pirro M. (2014). MiR128 up-regulation correlates with impaired amyloid β(1-42) degradation in monocytes from patients with sporadic Alzheimer’s disease. Neurobiol. Aging 35 (2), 345–356. 10.1016/j.neurobiolaging.2013.08.003 24064186

[B100] Tort F. Zieger K. Guldberg P. Sehested M. Bartkova J. Hor Z. (2005). DNA damage response as a candidate anti-cancer barrier in early human tumorigenesis. Nature 434, 864–870. 10.1038/nature03482 15829956

[B101] Tosca E. M. Ronchi D. Facciolo D. Magni P. (2023). Replacement, reduction, and refinement of animal experiments in anticancer drug development: the contribution of 3D *in vitro* cancer models in the drug efficacy assessment. Biomedicines 11 (4), 1058. 10.3390/biomedicines11041058 37189676 PMC10136119

[B102] Velimezi G. Liontos M. Vougas K. Roumeliotis T. Bartkova J. Sideridou M. (2013). Functional interplay between the DNA-damage- response kinase ATM and ARF tumour suppressor protein in human cancer. Nat. Cell Biol. 15 (8), 967–977. 10.1038/ncb2795 23851489

[B103] Verma D. K. Seo B. A. Ghosh A. Ma S. X. Hernandez-Quijada K. Andersen J. K. (2021). Alpha-synuclein preformed fibrils induce cellular senescence in Parkinson’s disease models. Cells 10, 1694–1697. 10.3390/cells10071694 34359864 PMC8304385

[B104] Vermunt M. W. Zhang D. Blobel G. A. (2019). The interdependence of gene-regulatory elements and the 3D genome. J. Cell Biol. 218 (1), 12–26. 10.1083/jcb.201809040 30442643 PMC6314554

[B105] Vicencio J. M. Galluzzi L. Tajeddine N. Ortiz C. Criollo A. Tasdemir E. (2008). Senescence, apoptosis or autophagy? When a damaged cell must decide its path - a mini-review. Gerontology 54 (2), 92–99. 10.1159/000129697 18451641

[B106] Vincent I. Rosado M. Davies P. (1996). Mitotic mechanisms in Alzheimer’s disease? J. Cell Biol. 132 (3), 413–425. 10.1083/jcb.132.3.413 8636218 PMC2120731

[B107] Vitek M. P. Araujo J. A. Fossel M. Greenberg B. D. Howell G. R. Rizzo S. J. S. (2020). Translational animal models for alzheimer’s disease: an alzheimer’s association business consortium think tank. Alzheimer’s Dement. Transl. Res. Clin. Interv. 6 (1), 121144–e12212. 10.1002/trc2.12114 PMC7798310 33457489

[B108] Vousden K. H. Lane D. P., (2007). p53 in health and disease. Nat. Rev. Mol. Cell Biol. 8, 275–283. 10.1038/nrm2147 17380161

[B109] Vousden K. H. Prives C. (2009). Blinded by the light: the growing complexity of p53, Cell 3, pp. 413–431. 10.1016/j.cell.2009.04.037 19410540

[B110] Wang C. Jurk D. Maddick M. Nelson G. Martin-ruiz C. Von Zglinicki T. (2009). DNA damage response and cellular senescence in tissues of aging mice. Aging Cell 8, 311–323. 10.1111/j.1474-9726.2009.00481.x 19627270

[B111] Wang S. Ke S. Wu Y. Zhang D. Liu B. He Y. H. (2021b). Functional network of the long non-coding RNA growth arrest-specific transcript 5 and its interacting proteins in senescence. Front. Genet. 12, 1–12. 10.3389/fgene.2021.615340 PMC7987947 33777096

[B112] Wang W. Zheng Y. Sun S. Li W. Song M. Ji Q. (2021a). A genome-wide CRISPR-based screen identifies KAT7 as a driver of cellular senescence. Sci. Transl. Med. 13 (575), eabd2655. 10.1126/SCITRANSLMED.ABD2655 33408182

[B113] Wilcock D. M. (2012). Neuroinflammation in the aging down syndrome brain; Lessons from Alzheimer’s disease. Curr. Gerontol. Geriatr. Res. 2012, 170276. 10.1155/2012/170276 22454637 PMC3290800

[B114] Wood J. A. Wood P. L. Ryan R. Graff-Radford N. R. Pilapil C. Robitaille Y. (1993). Cytokine indices in Alzheimer's temporal cortex: no changes in mature IL-1 beta or IL-1RA but increases in the associated acute phase proteins IL-6, alpha 2-macroglobulin and C-reactive protein. Brain Res. 629 (2), 245–252. 10.1016/0006-8993(93)91327-O 7509248

[B115] Xu M. Tchkonia T. Ding H. Ogrodnik M. Lubbers E. R. Pirtskhalava T. (2015). JAK inhibition alleviates the cellular senescence-associated secretory phenotype and frailty in old age. Proc. Natl. Acad. Sci. U. S. A. 112 (46), E6301–E6310. 10.1073/pnas.1515386112 26578790 PMC4655580

[B116] Yates S. C. Zafar A. Rabai E. M. Foxall J. B. Nagy S. Morrison K. E. (2015). The effects of two polymorphisms on p21cip1 function and their association with Alzheimer’s disease in a population of European descent. PLoS One 10 (1), 01140500–e114123. 10.1371/journal.pone.0114050 PMC4308198 25625488

[B117] Yoon S. Y. Kim D. H. (2016). Alzheimer’s disease genes and autophagy. Brain Res. 1649, 201–209. 10.1016/j.brainres.2016.03.018 27016058

[B118] Zare-shahabadi A. Masliah E. Johnson G. V. W. Rezaei N. (2015). Autophagy in alzheimer’s disease. Rev. Neurosci. 26 (4), 385–395. 10.1515/revneuro-2014-0076 25870960 PMC5039008

[B119] Zeng T. Ni H. Yu Y. Zhang M. Wu M. Wang Q. (2019). BACE1-AS prevents BACE1 mRNA degradation through the sequestration of BACE1-targeting miRNAs. J. Chem. Neuroanat. 98, 87–96. 10.1016/j.jchemneu.2019.04.001 30959172

[B120] Zhang R. Chen W. Adams P. D. (2007). Molecular dissection of formation of senescence-associated heterochromatin foci. Heterochromatin Foci ᰔ † 27 (6), 2343–2358. 10.1128/MCB.02019-06 PMC1820509 17242207

[B121] Zhang R. Poustovoitov M. V. Ye X. Santos H. A. Chen W. Daganzo S. M. (2005). Formation of MacroH2A-containing senescence-associated heterochromatin foci and senescence driven by ASF1a and HIRA. Dev. Cell 8, 19–30. 10.1016/j.devcel.2004.10.019 15621527

[B122] Zhang W. Zhao H. Wu Q. Xu W. Xia M. (2018). Knockdown of BACE1-AS by siRNA improves memory and learning behaviors in Alzheimer’s disease animal model. Exp. Ther. Med. 16 (3), 2080–2086. 10.3892/etm.2018.6359 30186443 PMC6122303

[B123] Zou L. Elledge S. J. (2003). Sensing DNA damage through ATRIP recognition of RPA-ssDNA complexes. Science 1542. 10.1126/science.1083430 12791985

